# Comparing enhancements to well-child visits in the prevention of obesity: ENCIRCLE cluster-randomized controlled trial

**DOI:** 10.1186/s12889-022-14827-w

**Published:** 2022-12-26

**Authors:** Lisa Bailey-Davis, Amy M. Moore, Melissa N. Poulsen, David A. Dzewaltowski, Stacey Cummings, Laina R. DeCriscio, Jennifer Franceschelli Hosterman, Daniel Huston, H. Lester Kirchner, Shawnee Lutcher, Carolyn McCabe, Gregory J. Welk, Jennifer S. Savage

**Affiliations:** 1 Department of Population Health Sciences, Geisinger, 100 N Academy Ave, Danville, PA 17822 USA; 2 Center for Obesity & Metabolic Research, Geisinger, 100 N Academy Ave, Danville, PA 17822 USA; 3 Center for Childhood Obesity Research, The Pennsylvania State University, 129 Noll Laboratory, University Park, PA 16802 USA; 4 College of Public Health, University of Nebraska Medical Center, 984365 Nebraska Medical Center, Omaha, NE 68198 USA; 5 Department of Pediatrics, Geisinger, 100 N Academy Ave, Danville, PA 17822 USA; 6 Health and Wellness, Steele Institute, Geisinger, 100 N Academy Ave, Danville, PA 17822 USA; 7 Departments of Internal Medicine and Pediatrics, Geisinger, 100 N Academy Ave, Danville, PA 17822 USA; 8 Department of Kinesiology, Iowa State University, 103E Forker, 534 Wallace Rd, Ames, IA 50011 USA

**Keywords:** Preschool-age, Rural; low-income, Obesity, Primary care, Patient centered outcome

## Abstract

**Background:**

Obesity disproportionally impacts rural, lower-income children in the United States. Primary care providers are well-positioned to engage parents in early obesity prevention, yet there is a lack of evidence regarding the most effective care delivery models. The ENCIRCLE study, a pragmatic cluster-randomized controlled trial, will respond to this gap by testing the comparative effectiveness of standard care well-child visits (WCV) versus two enhancements: adding a patient-reported outcome (PRO) measure (PRO WCV) and PRO WCV plus Food Care (telehealth coaching and a grocery store tour).

**Methods:**

A total of 2,025 parents and their preschool-aged children (20–60 months of age) will be recruited from 24 Geisinger primary care clinics, where providers are randomized to the standard WCV, PRO WCV, or PRO WCV plus Food Care intervention arms. The PRO WCV includes the standard WCV plus collection of the PRO—the Family Nutrition and Physical Activity (FNPA) risk assessment—from parents. Parents complete the PRO in the patient-portal or in the clinic (own device, tablet, or kiosk), receive real-time feedback, and select priority topics to discuss with the provider. These results are integrated into the child’s electronic health record to inform personalized preventive counseling by providers. PRO WCV plus Food Care includes referrals to community health professionals who deliver evidence-based obesity prevention and food resource management interventions via telehealth following the WCV. The primary study outcome is change in child body mass index z-score (BMIz), based on the World Health Organization growth standards, 12 months post-baseline WCV. Additional outcomes include percent of children with overweight and obesity, raw BMI, BMI50, BMIz extended, parent involvement in counseling, health behaviors, food resource management, and implementation process measures.

**Discussion:**

Study findings will inform health care systems’ choices about effective care delivery models to prevent childhood obesity among a high-risk population. Additionally, dissemination will be informed by an evaluation of mediating, moderating, and implementation factors.

**Trial registration:**

ClinicalTrials.gov identifier (NCT04406441); Registered May 28, 2020.

## Background

Obesity often begins early in life, with recent trends showing increasingly early onset of disease. National data from 2017–2018 suggest that 13.4% of preschool-age children (aged 2–5 years) in the United States (US) have obesity, an increase from 8.4% in 2011–2012 [1]. Risk factors for disparities in obesity prevalence among children include rural residence, lower household income, and community-level socioeconomic deprivation [2–5]. The preschool years are a critical period for prevention interventions, as rapid gains in body mass index (BMI) during this period can lead to obesity and substantial health burdens, including poor cardiovascular and metabolic outcomes characterized by high blood pressure, dyslipidemia, and insulin resistance [6–8], and other adverse physical health effects [9, 10] during childhood. Children with obesity also report detrimental social and emotional health outcomes, including lower self-esteem and health-related quality of life when compared to children with normal weight [11].

Adverse effects of childhood obesity persist over time, leading to a substantial public health burden. Longitudinal studies show that for 60 to 90% of preschool-aged children with obesity, the condition persisted into adolescence [12, 13] and adulthood [2, 14]. Obesity that persists into adulthood often has negative effects on health [12–14] and has a significant impact on overall population health. Based on the prevalence of obesity among US children aged 2–19 years in 2016, and assuming no changes in secular trends, 57% of the US population is projected to experience obesity before age 35 years**,** and thus may experience obesity-related comorbidities [15].

Addressing complex multifactorial public health challenges such as childhood obesity requires primary prevention approaches [16]. Well-child visits (WCV) in primary care clinics provide a sustainable model for intervention delivery, particularly because parents and caregivers (hereafter “parents”) of young children value and trust feedback from pediatricians [17]. Primary care providers (PCPs) are on the frontlines of obesity prevention, yet clinical preventive care has had limited success in preventing obesity during childhood. Nearly all children attend WCVs, yet the prevalence of preschool-aged children with obesity has increased and remains high [1]. Although PCPs are well-positioned to follow clinical preventive guidelines to engage parents and provide referrals to community services [18, 19], progress has been hindered by a lack of evidence regarding effective models to prevent childhood obesity during the preschool years [20, 21].

In the context of clinical care, collection of patient-reported outcome (PRO) measures offers a promising strategy to enhance patient-centered care by engaging parents in discussions related to preventive care. PRO measures are a standardized method to collect information directly from patients on their experiences, perceptions, or beliefs relative to a disease or health outcome [22, 23]. With the advancement of health information technology, PRO measures can be implemented in easy-to-use formats for patients, often via an online patient portal or a tablet in the clinic waiting room. Responses can be integrated into patients’ electronic health record (EHR), allowing for automatic scoring, efficient review by providers, and immediate implementation into patient-centered care [24]. WCVs can be enhanced with PRO measures to engage parents in self-assessment of behaviors, practices, and home environments that are associated with obesity and predict risk before obesity is established [25–27]. Our previous work demonstrated that using a validated questionnaire, the Family Nutrition and Physical Activity (FNPA) [28, 29], systematically collected at WCVs in a large health system, prevented obesity among preschool-age children [30]. Other studies have demonstrated that family-centered health coaching with parents related to nutrition and physical activity and referrals to community resources, such as grocery store tours, following WCVs are efficacious approaches to preventing childhood obesity [31, 32] and promoting food resource management to help reduce food insecurity among low-income families [33]. Taken together, evidence suggests that enhancing WCVs with PRO measures, health coaching, and community referrals can prevent childhood obesity, although evidence is lacking about which models are most effective in real-world settings.

The current study will respond to this gap by comparing two enhancements to standard WCVs among a high-risk population of preschool-age children receiving care at Geisinger, a large integrated health system in a largely rural area of Pennsylvania. The ENCIRCLE (patiEnt-cliNic-Community Integration to prevent obesity in Rural preschool ChiLdrEn) study was designed to test the comparative effectiveness of standard WCVs versus two enhancements (PRO WCV and PRO WCV plus Food Care) on obesity prevention among preschool-aged children at risk of obesity. PRO WCV includes collection of the FNPA risk assessment from parents. PRO WCV plus Food Care includes the FNPA as well as referrals to community health professionals who deliver evidence-based obesity prevention and food resource management interventions via telehealth following WCVs. We hypothesize that children in PRO WCV and PRO WCV plus Food Care will have a lower change in BMI z-scores (BMIz) at 12 months post-baseline compared to the standard WCV. We will also examine the percent of children with overweight and obesity at 12 months post-baseline and difference in raw BMI, BMI50, and BMIz extended, as well as the interventions’ effects on parents’ perceptions of involvement in preventive counseling and other home and parenting factors. Secondarily, multilevel mediators and moderators of intervention effectiveness will be examined. In the tradition of Type I hybrid effectiveness-implementation research [34], this study also aims to identify factors that influence implementation at the parent, PCP, and clinic levels through an evaluation of the reach, adoption, implementation, and maintenance of each intervention arm using the RE-AIM framework [35].

## Methods/design

### Study overview

**Fig. 1:**
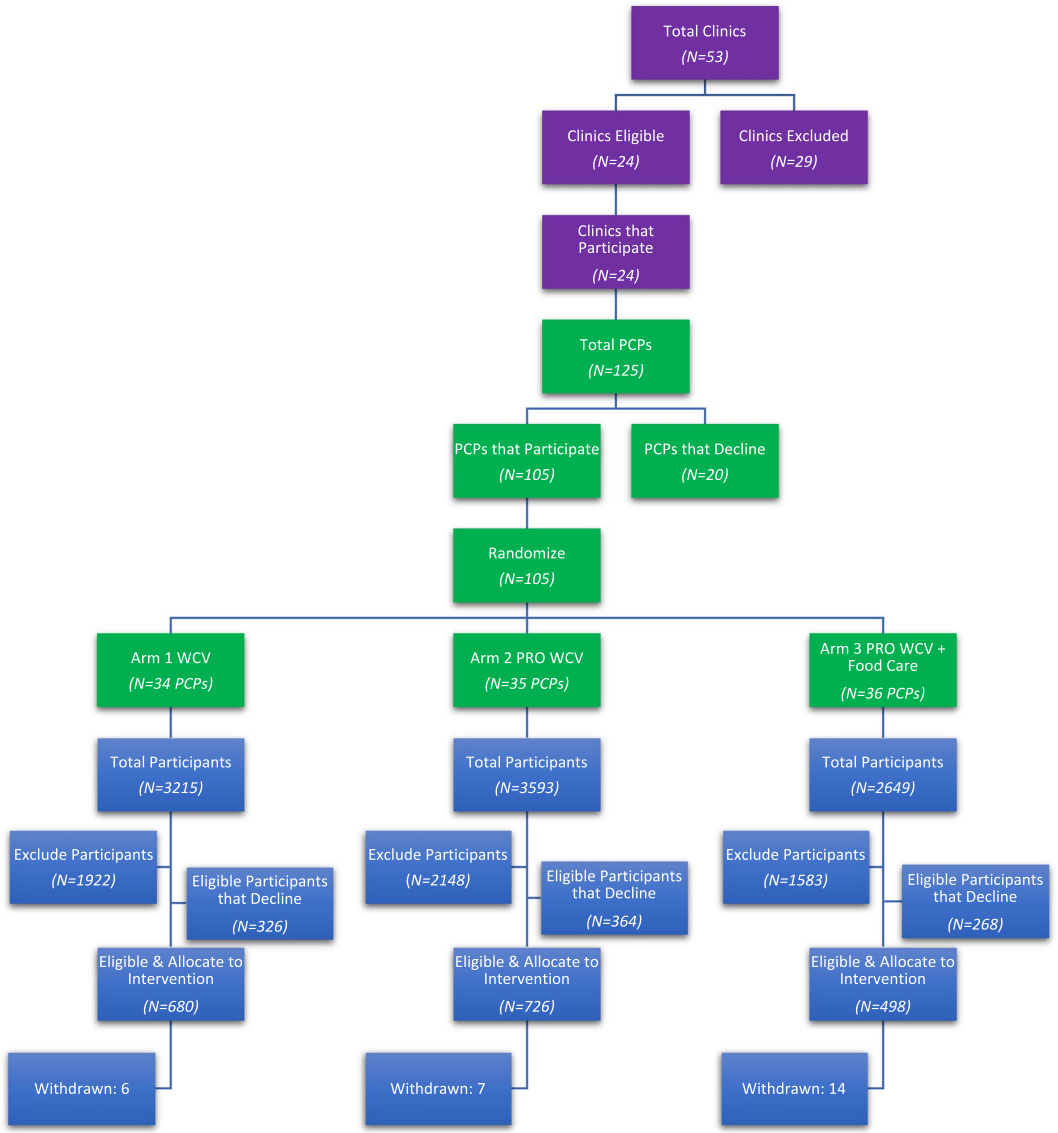
ENCIRCLE CONSORT diagram

The ENCIRCLE study is a pragmatic, cluster-randomized controlled trial conducted in 24 Geisinger primary care clinics in central and northeast Pennsylvania. These clinics are predominantly located in rural areas and serve socioeconomically diverse patients, and thus reach families at high risk of health disparities. A total of 105 PCPs from family medicine (*n* = 51) and pediatric (*n* = 54) clinics were randomized to one of the following intervention arms: WCV, PRO WCV, or PRO WCV plus Food Care. Randomization occurs at the provider level; thus PCPs within the same clinic may be in different study arms. The WCV arm is consistent with clinical guidelines [18, 19] and includes BMI screening and brief preventive counseling by the PCP. The PRO WCV and PRO WCV plus Food Care arms add the FNPA risk assessment to the WCV [25, 28, 29]. In the PRO WCV plus Food Care arm, parents also receive referrals to community health professionals [32] who deliver evidence-based obesity prevention [31] and food resource management [32, 33] interventions via telehealth [32]. Telehealth delivery has been fortuitous as the study began in March 2020, coinciding with the US emergence of the COVID-19 pandemic and the rapid transition to telemedicine to minimize virus transmission. Figure 1 presents the ENCIRCLE CONSORT diagram. This study was approved by Geisinger’s Institutional Review Board and is registered with ClinicalTrials.gov (NCT04406441).

### Eligibility

#### Primary care clinics & PCPs

Pediatric and family medicine primary care clinics were eligible for study participation if they had one or more PCPs with at least 32 WCVs annually for preschool-aged children with BMI-for-age and -sex ≥ 50^th^ percentile and had no prior exposure to PRO WCV. PCPs working within these clinics were eligible for study participation if they conduct WCVs. Prior to randomization, eligible PCPs were stratified based on the type of clinic in which they practice (family medicine versus pediatrics) and caseload (number of annual WCVs conducted among the target population). These criteria resulted in three strata: all of pediatrics, family medicine with a low volume (< 32 WCV/year), and family medicine with a higher volume (≥ 32 WCV/year). Participating PCPs were randomized to one of three study arms and all age-eligible patients seen by these PCPs receive the care model aligned with the study arm. In other words, all patients seen by a PCP in a PRO WCV arm are asked to complete FNPA regardless of study enrollment. Systematic implementation of PRO at the PCP-level facilitates clinical workflow and blinds the PCP to parent–child study participation to reduce bias.

#### Parent–child Dyads

Eligible patients are between ages 20 and 60 months who have a BMI-for-age and -sex ≥ 50^th^ percentile. Children who regularly attend their annual WCV via telemedicine, have a preexisting medical condition that would impact study participation (e.g., type 1 diabetes, cancer, and major developmental delays), or have a sibling participating in the study are excluded to minimize risk and avoid potential confounding. Eligible parents are ≥ 18 years of age, English-speaking, from lower-income households, and with no plans to relocate outside of their current provider’s service area or change pediatric providers in the next two years. Households are considered lower income if they are eligible for or receiving Special Supplemental Nutrition Program for Women, Infants and Children (WIC), Supplemental Nutrition Assistance Program (SNAP), Temporary Assistance for Needy Families (TANF), Medicaid, Children’s Health Insurance Program (CHIP), or National School Lunch or Breakfast Program (including curbside pick-up during COVID-19); or if they screen positive for household food insecurity [36].

#### Recruitment & informed consent

Potentially eligible patients who recently completed or have an upcoming annual WCV with a participating PCP are identified and screened using an EHR system query based on study inclusion and exclusion criteria. Parents of potentially eligible patients are simultaneously sent a recruitment letter, an email (if available), a Geisinger online patient portal message (if available), and a text message (if available and consented to receive from the health system) that provides information regarding study participation, how to opt out, and a study URL for self-screening. Both the recruitment letter and email encourage parents to enroll in the patient portal to facilitate scheduling and completion of pre-visit questionnaires (consistent with standard care). Additional strategies are used to reach parents, including distribution of a study flyer and participant story through social media (Facebook), in-person and electronic flyer distribution by community health partners (i.e., HeadStart, Nurse Family Partnership Program, and WIC), flyer distribution by snowball email (participants refer friends), and in-clinic recruitment. All strategies encourage parents to enroll in the study using the study URL or QR-code provided. Specifically, parents can complete screening, access study information, contact the study team, and electronically consent via the link/code. Ten days after mailing the recruitment letter and/or three days after sending the e-mail, study team members contact parents who have yet to opt-out or respond using the link/code to assess interest. Once contacted by phone, the study team discusses the study with the parent, and if interested, obtains verbal informed consent to participate in data extraction from their child’s EHR, online survey data collection at baseline and 6- and 12-months post-baseline assessment, as well as participation in the community telehealth coaching intervention (as applicable). Parent–child dyads in each study arm schedule and attend their usual annual WCV. Parents in the PRO WCV and PRO WCV plus Food Care are asked to complete the FNPA as standard care. Parents in PRO WCV plus Food Care are referred by the study team to community health providers. Parent–child dyads are assigned a study identifier to protect confidentiality.

### Description of ENCIRCLE intervention components

**Fig. 2:**
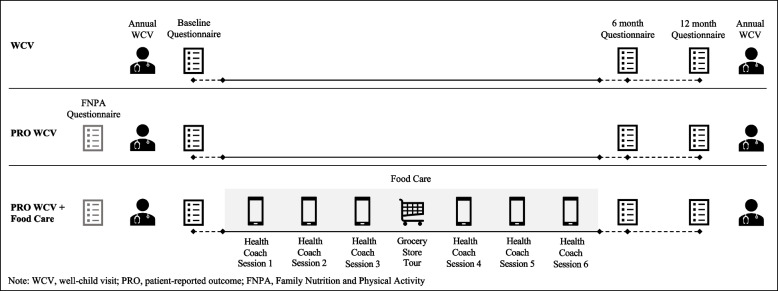
Overview of ENCIRCLE by study arm

**Figure ****2** provides an overview of ENCIRCLE components by study arm.

### Well-child visit (WCV) arm

The standard WCV is consistent with clinical guidelines [18, 19]. PCPs conduct face-to-face visits that include a review of patient health history, age-appropriate measurements (height/length, weight, and blood pressure), sensory and developmental screenings, physical exams, immunizations, and brief preventive counseling. WCVs also include a BMI assessment and verbal inquiry by the PCP about obesity risk factors, such as child nutrition and physical activity behaviors, to inform brief preventive counseling and promote maintenance of healthy weight.

All primary care clinic staff are trained and receive booster sessions on anthropometric methods for pediatric height and weight measurement. Training is also provided for entering measurements into the EHR system to generate sex-specific BMI-for-age percentiles to identify children by weight status to guide preventive counseling.

### Patient-reported outcome (PRO WCV) arm

PCPs randomized to the PRO WCV arm utilize the EHR system to enhance the WCV by collecting and integrating a PRO measure in the patient’s EHR and clinical workflow [30]. The FNPA is a validated 20-item PRO measure designed to identify behavioral and environmental risk factors for obesity during childhood [28, 29, 37]. The FNPA takes 2–3 min to complete and includes questions identifying nutrition, physical activity, screen time, and sleep risk behaviors to inform patient-centered preventive counseling [26]. As standard care, 10 days prior to the scheduled WCV, parents receive an automated email with a link to the FNPA, which is hosted in the patient portal. If the FNPA is incomplete when the patient presents for the WCV, clinic staff implement a variety of workflow models to collect data. Parents can digitally complete the FNPA in the waiting area or in the exam room. The latter option is useful when patients are roomed quickly to minimize risk of COVID-19 transmission. Parents can use their own device to authenticate into the patient portal to complete the FNPA. Alternatively, staff may offer a sanitized tablet or direct parents to a kiosk or use a touchscreen in the exam room. Upon FNPA completion, parents receive real-time patient-centered feedback regarding risk reduction strategies and are prompted to identify priority topics that they would like to discuss with their child’s PCP to facilitate agenda-setting. Providing real-time feedback and obtaining top concerns from parents was recommended by the Geisinger Patient Advisory Council on Obesity (PACO), which includes health system, community partners, and researchers, and has been beneficial in other obesity prevention studies [38].

The PCP views the FNPA results in the child’s EHR. Results are displayed succinctly in a table embedded within a WCV template. The parent’s priority topics are displayed at the top of the table followed by FNPA results, with the most obesogenic responses in bold font to efficiently orient the PCP to high-risk behaviors. Smart features within EPIC® EHR facilitate PCP documentation of nutrition and physical activity counseling and the selection of educational materials.

All Geisinger PCPs were trained and receive booster trainings on the FNPA. However, PCPs in the WCV arm are considered “wait-listed,” with FNPA implementation delayed until study completion to avoid the risk of contamination between study arms. Trainings were delivered remotely by the study team’s lead clinical investigator and delivered synchronously during routine clinical staff meetings or asynchronously as a recorded video viewed at the PCP’s convenience. The recorded video option was added in 2020 to facilitate clinic adoption in the context of national health care staffing shortages during the COVID-19 pandemic. Written “fast facts” are also provided as a point-of-care reference. A clinic-level audit of FNPA completion rates is assessed quarterly, with feedback provided to clinic staff with PCPs in PRO arms. To enhance adoption of the clinical workflow for FNPA, clinics with 75% FNPA completion or a 25% improvement in FNPA completion over the prior quarter are acknowledged with a staff boxed lunch. The audit/feedback is an aggregate report, without stratification by patient study participation.

### PRO WCV plus Food Care arm

Following the PRO WCV, parents in the PRO WCV plus Food Care arm are contacted by phone by a study team member and referred to the community health coaches employed by the Geisinger Wellness Program. A trained health coach delivers an evidence-based obesity prevention intervention adapted from the Choose Health LA: Parent Training Program [31, 39] for telehealth delivery. The study team adapted the program from monthly in-person small group sessions to monthly remote one-on-one sessions delivered by the health coach across the 6-month intervention period. Social cognitive theory [40], adult learning theory [41], and motivational interviewing [42] informed adaptations. The program provides parents with age-appropriate evidence-based nutrition, physical activity, and parenting guidance. Each session includes behavior change goal setting to help parents self-select and achieve goals that matter most to them. Additionally, the Food Care arm includes a referral for parents to participate in a grocery shopping program adapted from Cooking Matters at the Store® [43] for telehealth delivery. The program includes a single, one-on-one or group telehealth session delivered by trained in-store nutritionists that provides parents with actionable strategies related to healthy grocery shopping on a budget, including purchasing produce and comparing unit prices. The Parent Training Program has been shown to prevent obesity among preschool-aged children [31] and Cooking Matters® has demonstrated improved food resource management to help reduce food insecurity among low-income families [33]. Table 1 provides an overview of the PRO WCV plus Food Care timeline and components.

**Table 1:** PRO WCV plus Food Care timeline and intervention components^a^

Session (Timing)	Provider	Duration (Setting)	Session Names and Components
Session 1 (month 1)	Health Coach	60-min (virtual)	• nutrition: changing how we serve food • parenting: power of praise • components: topic review, goal setting
Session 2 (month 2)	Health Coach	20-min (virtual)	• nutrition: healthy eating for your child • parenting: giving commands that work • components: topic review, goal setting
Session 3 (month 3)	Health Coach	20-min (virtual)	• nutrition: reading nutrition labels • parenting: daily routines • components: topic review, goal setting
Store Tour (month 3–4)	Nutritionists	45–60 min (virtual)	• general: healthy shopping on a budget • components: purchasing produce, comparing unit prices, reading food labels, identifying whole grains
Session 4 (month 4)	Health Coach	20-min (virtual)	• nutrition: healthy eating and shopping • parenting: weekly routines • components: topic review, goal setting
Session 5 (month 5)	Health Coach	20-min (virtual)	• nutrition: sugar-sweetened beverages and activity • parenting: ignoring unwanted behaviors • components: topic review, goal setting
Session 6 (month 6)	Health Coach	20-min (virtual)	• nutrition: healthy snacks and celebrations • parenting: enforcing rules • components: topic review, goal setting

^a^ ENCIRCLE Parent Training Program (Sessions 1- 6) adapted from Choose Health LA: Parent Training Program for telehealth delivery. Store Tour adapted from Cooking Matters at the Store® for virtual delivery

The intervention health coaches were trained and receive monthly booster trainings on the Parent Training Program and basic counseling skills (e.g., developing rapport and active listening). The initial 6-h training was conducted virtually across two sessions by a doctoral-level registered dietitian. Grocery store nutritionists were trained and received booster training on the adapted Cooking Matters at the Store® program. The initial 3-h training was conducted virtually by a doctoral-level registered dietitian and a 1-h booster training was conducted prior to implementation.

### Measures

Questionnaires are collected and managed using REDCap [44, 45] electronic survey system hosted at Geisinger. Parents are emailed or texted a link to the electronic survey system for completion. Upon parent request, hardcopies of questionnaires with a prepaid return envelope are mailed. Table 2 includes an overview of anthropometrics and questionnaires collected at each study time point.

**Table 2:** ENCIRCLE anthropometrics and questionnaires by time point

	**Time Point**^a^				
	**Screening**	**Baseline**	**6 months**	**12 months**	**24 months**
**Caregiver-child Measures**					
Child Anthropometrics^b^	X	X		X	
Demographics^b,c^	X	X			
Household food security [36]		X	X	X	
Perceived involvement in pediatric care [46]		X	X	X	
Obesity prevention attitudes and intentions [47]		X	X	X	
Perception of neighborhood [48]		X	X	X	
Food parenting practices [49, 50]		X	X	X	
Food resource management [33]		X	X	X	
Child dietary behaviors [51]		X	X	X	
Child physical activity [52]		X	X	X	
Child sleep [53]		X	X	X	
Child screen time [54]		X	X	X	
Child life satisfaction [55]		X	X	X	
Program satisfaction^d^			X		
**PCP & Health Coach Measures**					
Demographics		X			
Organizational climate [56–58]		X		X	X
Obesity prevention attitudes and intentions [59]		X		X	X
Preventive counseling for obesity self-efficacy [60, 61]		X		X	X
Intervention implementation^e^			X		

^a^ Time point is specific to group

^b^ Retrieved from electronic health record (EHR)

^c^ Caregiver demographics collected at screening or baseline; child demographics retrieved from EHR

^d^ Completed by caregivers in PRO WCV plus Food Care following health coach sessions 1 and 6

^e^ Completed by the health coach following session 1–6

### Anthropometric measures

Child height and weight are measured and recorded using standardized procedures during WCVs by trained clinic staff. Height is measured to the nearest 0.1 cm using a stadiometer (SECA 264) and weight is measured to the nearest 0.1 kg using a calibrated digital scale (Healthometer 599KL). Sex-specific BMI-for-age percentiles are calculated in the EHR system to identify children by weight status: normal weight (> 5^th^ and < 85^th^), overweight (≥ 85^th^ and < 95^th^), obese (≥ 95^th^ and < 99^th^), and severely obese (≥ 99^th^). Anthropometric data are stored in the child’s EHR.

### Questionnaires

#### Parent–child dyad

Information collected at baseline from parents includes self-reported age, biological sex, race/ethnicity, height and weight status, relationship to child, educational level, annual household income, and employment status. Parents also complete the validated 6-item US Department of Agriculture Food Security Scale [36] and report enrollment in federally-funded assistance programs (e.g., WIC, SNAP, and TANF).

Parents are asked to complete questionnaires at three time points (baseline and 6- and 12-months post-baseline assessment). Parents self-report their involvement in their child’s pediatric care, obesity prevention attitudes and intentions, food parenting practices, food resources management, and child health-related behaviors (e.g., dietary behaviors, physical activity, sleep, and screen time). In addition, parents in the PRO WCV plus Food Care arm complete a 10-item satisfaction questionnaire after their first and last health coaching session. Parents receive gift cards for completing questionnaires ($50 at baseline, 6, and 12 months), and, to aid in retention, a bonus of $50 if questionnaires are completed at all three time points.

#### PCP and health coach

Participating PCPs and health coaches complete questionnaires at three time points (baseline and 12- and 24-months post-baseline). Questionnaires include items related to organizational climate, obesity prevention attitudes and intentions, and self-efficacy for preventive obesity counseling. In addition, following each session with parents, the health coach completes a questionnaire related to intervention implementation in order to track fidelity to intervention content.

#### Implementation measures

The RE-AIM framework is used to describe and evaluate elements of the study related to external validity [35]. The RE-AIM framework is widely used in implementation studies and focuses on aspects of the implementation process that can facilitate or constrain success in achieving the intended impact of an intervention [62]. This is especially important in the present study which takes place within multiple systems (i.e., communities, a health system, clinics, and providers). Table 3 shows planned RE-AIM outcome measures. Adaptations to the study protocol are documented using the Framework for Reporting Adaptations and Modifications-Enhanced [63] to provide contextual and process data to support interpretation of study findings and to guide future implementation.

**Table 3:** RE-AIM outcome measures

RE-AIM Dimension and Description	Measures	Data sources
**Reach** *Degree to which target population is exposed to intervention*	• Number of eligible caregivers/children • % of caregivers/children who consent to participate • % of caregivers/children who complete study follow-up • Representativeness of caregivers/children who enrolled compared to overall population of screened and eligible caregivers/children • Reasons why caregivers/children decline participation	• Study database derived from EHR clinical data and study records • Caregiver questionnaire implemented via REDCap at baseline • Brief non-participant survey administered via phone or online for caregivers who decline participation
**Effectiveness** *Intervention success in changing patient outcomes*	• Improvement in primary and secondary outcomes by study arm • Heterogeneity of effects across caregiver/children subgroups • Attrition by study arm and by caregiver/children subgroups	• Analytic plan for the primary and secondary outcomes is discussed in Statistical Analyses
**Adoption** *Degree to which interventions are taken up by decision makers (e.g., clinics, providers)*	• % of PCPs adopting the intervention by study arm • Representativeness of PCPs who adopt the Intervention compared to the overall population of providers eligible to participate	• PCP attendance at a virtual study training at baseline • Clinic leaders via interview
**Implementation** *Degree to which intervention was delivered as intended, adaptations, and drivers of adaptations*	Across Implementation Agents •Implementation input and processes, including challenges to implementation noted by study team and stakeholders • Adaptations to intervention components and study protocol • Decision-maker who initiated adaptation and reasons for adaptation Agent Implementation • PCPs’ and health coach’s self-reported barriers to completing intervention components • % and characteristics of participating PCPs who utilize FNPA (arms 2 and 3) Caregiver Receipt of Intervention • Caregivers’ self-reported barriers to participating in intervention components • % and characteristics of participating caregivers who complete FNPA (arms 2 and 3) • % and characteristics of participating caregivers who complete Food Care sessions and who attend the virtual grocery store tour (arm 3)	• Study records such as meeting minutes • Adaptation tracking spreadsheet • PCP study questionnaires implemented via REDCap • Health coach questionnaire implement via REDCap • Caregiver questionnaires implemented via REDCap
**Maintenance** *Degree to which the intervention is sustained across settings and patients*	• Plan and commitment from health system and community partners to continue or expand intervention • % of PCPs maintaining use of FNPA in 12 months following intervention completion • % of participating caregivers who return to the clinic for a 12-month follow-up WCV • % of caregivers completing FNPA at 12 months (arms 2 and 3)	• Study database derived from EHR clinical data and study records • Stakeholder discussions with health system leaders and community partners

#### Patient and stakeholder engagement

Three primary stakeholder groups were engaged in study planning: parents of pediatric patients, PCPs, and healthcare administrators. Four PACO partners agreed to participate in quarterly meetings along with other stakeholders (clinical leaders and health coach supervisor). Ongoing input from stakeholders guides recruitment and retention, implementation, and dissemination efforts, and stakeholder perspectives are valuable contributions to the decision-making process.

### Sample size and statistical analysis

#### Sample size

The primary outcome for this study is change in child BMIz at 12 months post-baseline WCV. Preliminary data comparing change in child BMIz between WCV versus PRO WCV showed a change of 0.05 units corresponding to an effect size of 0.20 [30]. To determine sample size, we used an effect size of 0.20, an overall significance level of 0.05 (0.025 for each comparison, i.e., WCV versus PRO WCV and WCV versus PRO WCV plus Food Care), an intra-cluster correlation coefficient at 0.005, and a coefficient of variation at 0.25. This study will have 80% power to detect an effect size of 0.20 or larger, with a total of 1,920 children across the three arms with 20 PCPs per arm and 32 children per PCP. The estimated power to detect an effect improves as the number of PCPs per cluster increases but is reduced with fewer children per PCP.

#### Statistical analyses

Descriptive statistics include means with standard deviations and medians with interquartile range for continuous variables, and frequency with percentages for categorical variables. Data will be summarized for enrolled patients and stratified by randomization arm and study time point.

### Primary aims: change in child BMIz at 12 months post-WCV

The primary outcome is change in child BMIz at 12 months post-baseline WCV, which will be compared between WCV versus PRO WCV and WCV versus PRO WCV plus Food Care. Intent-to-treat analysis principles will be followed. Linear mixed models will be used to examine group differences, accounting for the correlations between patients from the same PCP. Models will be adjusted for stratification factors used in the randomization scheme and any baseline variables found to differ across intervention arms. Differences in the percent of children with overweight and obesity at 12 months post-baseline WCV, raw BMI, BMI50, and BMIz extended, parent involvement in counseling, health behaviors, food resource management and household food security will be compared as secondary outcomes.

### Secondary aims: mediators and moderators of change in child BMIz at 12 months post-WCV

An evaluation of whether intervention effectiveness is mediated by PCP, caregiver, and child health behaviors will be conducted. Linear mixed models will be used to assess hypothesized mediators as outcome variables. Models will also include child BMIz as the outcome variable and each potential mediator as an independent variable. Any variable found to be associated with intervention and BMIz will be considered a potential mediator variable. Our candidate mediator variables are measured at both the patient (parent and child) and cluster levels (PCP). Multilevel structural equation models will be used to assess potential mediators and moderators. Candidate moderators include community socioeconomic deprivation, rurality, perception of neighborhood, and food access. Clinic organizational climate, household income, and transportation will also be evaluated as potential moderators of intervention effectiveness.

### Missing data

Several strategies will be used to minimize missing data including sending scheduling and appointment reminders to parents for the follow-up WCV in the patient’s EHR. However, missing data are inevitable in a prospective study due to dropout and nonresponse to study questionnaire items. Based on previous research, it is anticipated that no more than 7% of randomized participants will not receive allocated treatment and 10% of randomized participants will not complete the study [64, 65]. Before completing primary analyses, patterns of missingness will be characterized, and depending on the amount of missing data, a non-parametric missing data imputation method based on random forests will be used [66]. This method has been shown to perform as well as or better than more traditional methods of imputation, and it has the advantage of imputing both continuous and categorical data.

### Analyses to assess heterogeneity of treatment effects

Given that intervention effects may vary among patients, additional subgroup analyses will be performed by child sex (male, female) and baseline BMI weight status (overweight, obese).

### Analyses to assess external validity using the RE-AIM framework

Using measures based on the RE-AIM framework, intervention reach, effectiveness, adoption, implementation, and maintenance will be descriptively evaluated to inform translation and future scale-up and scale-out. The effectiveness component of RE-AIM will be addressed through the primary and secondary analysis aims described above, which include assessment of heterogeneity of effects across caregiver subgroups (e.g., food insecurity status). In addition, attrition will be evaluated by intervention arm and caregiver subgroups.

## Discussion

The ENCIRCLE study responds to a need for evidence regarding the most effective clinical care delivery models for obesity prevention during early childhood. Risk factors for disparities in obesity prevalence include rural residency, lower household income, and community-level socioeconomic deprivation [2–5]. PCPs and policymakers are aware of this disparity, yet rural children remain underrepresented in the literature. Findings from this pragmatic trial testing the comparative effectiveness of WCV versus two enhancements will inform care delivery models for rural, lower-income preschool-age children at risk for obesity. While both PRO measures (e.g., FNPA [30]) and Food Care components [31–33] have been effective in preventing obesity during childhood, the ENCIRCLE study is unique in comparing the effectiveness of these models versus WCV in rural primary care.

PCPs who conduct WCVs are well-positioned to follow clinical preventive guidelines [18, 19], yet providers face decisional dilemmas regarding allocating limited time and resources to nutrition and physical activity counseling. Enhancing WCVs with PRO measures, such as the parent-reported FNPA, will help PCPs quickly identify risk factors and provide real-time patient-centered feedback and referrals to parents of preschool-aged children at risk for obesity. Further enhancing WCVs with health coaching and community referrals will engage parents of preschool-aged children in patient-centered care to help parents improve health behaviors that are important to their family. Taken together, understanding the effects of these enhancements will offer insights into the most effective care delivery model for obesity prevention during early childhood.

Findings from the ENCIRCLE study will also inform dissemination strategies to best engage parents, primary care clinics, and PCPs in PRO measure collection, preventive counseling, and community referrals based on the RE-AIM framework [35, 62]. Despite the importance of examining intervention implementation, a systematic review of child dietary interventions with a parent component found a concerning lack of reporting on RE-AIM elements, particularly those related to external validity [67]. This lack of reporting on implementation constrains effective translation of interventions into primary care, thus limiting the potential population health impact of these interventions. This is particularly important given the challenges of initiating the study during the COVID-19 pandemic, when secular trends indicated significant increases in child BMI [68] and preventive care was disrupted as resources were redirected for mitigation efforts [69]. The resulting downturn in routine WCVs has been significant among preschool-age children and those who experience adverse social determinants of health [70].

Obesity disproportionally impacts rural, lower-income children in the US. PCPs serving rural families are well-positioned to engage parents in early obesity prevention, yet there is a lack of evidence regarding the most effective care delivery models. Testing the comparative effectiveness of enhancements to standard WCVs will provide evidence for which care delivery models are most effective in real-world settings, thus helping to disrupt disconcerting trends that show increasing prevalence of obesity during early childhood.

## Data Availability

The materials and/or data from the current study are available from the corresponding author upon request.

## Abbreviations

WCV: Well child visit
PRO: Patient-reported outcome
FNPA: Family Nutrition and Physical Activity
BMI: Body mass index
PCP: Primary care provider
EHR: Electronic health record
WIC: Special Supplemental Nutrition Program for Women, Infants and Children
SNAP: Supplemental Nutrition Assistance Program
TANF: Temporary Assistance for Needy Families
CHIP: Children’s Health Insurance Program
URL: Uniform resource locators
PACO: Patient Advisory Council on Obesity
